# Alpha-2-macroglobulin loaded microcapsules enhance human leukocyte functions and innate immune response

**DOI:** 10.1016/j.jconrel.2015.09.021

**Published:** 2015-11-10

**Authors:** Donata Federici Canova, Anton M. Pavlov, Lucy V. Norling, Thomas Gobbetti, Sandra Brunelleschi, Pauline Le Fauder, Nicolas Cenac, Gleb B. Sukhorukov, Mauro Perretti

**Affiliations:** a William Harvey Research Institute, Barts and the London School of Medicine, Queen Mary University of London, London, United Kingdom; b School of Engineering & Materials Science, Queen Mary University of London, London, United Kingdom; c Saratov State University, Saratov, Russia; d Department of Health Science, University of Eastern Piedmont, Novara, Italy; e MetaToul Lipidomics Facility, INSERM UMR1048, Toulouse, France; f INSERM UMR1043, Université Toulouse III Paul-Sabatier, Toulouse, France

**Keywords:** Alpha-2-macroglobulin, LbL microcapsules, Inflammation, Leukocyte activation, Phagocytosis

## Abstract

Synthetic microstructures can be engineered to deliver bioactive compounds impacting on their pharmacokinetics and pharmacodynamics. Herein, we applied dextran-based layer-by-layer (LbL) microcapsules to deliver alpha-2-macroglobulin (α2MG), a protein with modulatory properties in inflammation. Extending recent observations made with dextran-microcapsules loaded with α2MG in experimental sepsis, we focused on the physical and chemical characteristics of these microstructures and determined their biology on rodent and human cells. We report an efficient encapsulation of α2MG into microcapsules, which enhanced i) human leukocyte recruitment to inflamed endothelium and ii) human macrophage phagocytosis: in both settings microcapsules were more effective than soluble α2MG or empty microcapsules (devoid of active protein). Translation of these findings revealed that intravenous administration of α2MG-microcapsules (but not empty microcapsules) promoted neutrophil migration into peritoneal exudates and augmented macrophage phagocytic functions, the latter response being associated with alteration of bioactive lipid mediators as assessed by mass spectrometry. The present study indicates that microencapsulation can be an effective strategy to harness the complex biology of α2MG with enhancing outcomes on fundamental processes of the innate immune response paving the way to potential future development in the control of sepsis.

## Graphical abstract

**Figure:**
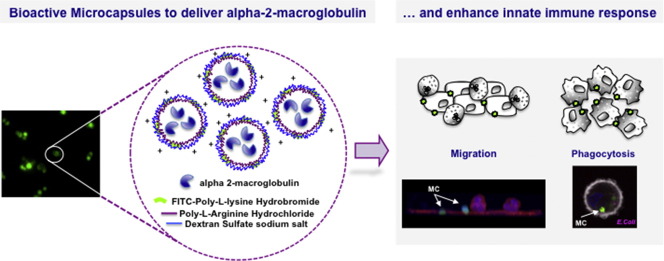

## 1 Introduction

A promising method to provide controlled, sustained delivery and release of proteins is the layer-by-layer (LbL) microencapsulation technique [1]. Several studies have focused on the construction of nano- and micro-capsules engineered as carriers for active compounds including, enzymes, nucleic acids, proteins and chemo-therapeutics. These protocols enact a drug-delivery system to carry controlled quantities of a therapeutic payload to a specific target site or tissue. Their main advantages are versatility, control over function and response-tailored to capsule structure. Multi-compartmental structure may allow inclusion of various compounds at defined doses in a single vesicle, altering their activity and accessibility to environment [2], [3], [4]. Extensive research of microcapsule (MC) delivery shows internalization by target cells without overt toxicity. Microcapsules made of biodegradable polymers can degrade over time to gradually release encapsulated compounds, a phenomenon reported both *in vivo* and *in vitro* [5]. All these characteristics make microcapsules a versatile delivery tool, amenable to the delivery of proteins that can modulate the inflammatory process.

Most proteins have short half-life when applied *in vivo*, requiring multiple administrations. Encapsulation often yields i) amelioration of bioactions, ii) enhancement of therapeutic efficacy by delivery to a specific tissue and iii) delivery across biological barriers. Among the mediators of the inflammatory process, the acute phase protein alpha-2-macroglobulin (α2MG) is of particular interest. α2MG acts as a protease inhibitor and carrier for several growth factors and cytokines, including TNF-α, IL-1β, IL-6 and TGF-β [6]. Activation of α2MG results in the entrapment of proteases with the entire complex now being able to bind to the low-density lipoprotein receptor like protein-1 (LRP-1; [7]), the α2MG receptor. Therefore, the α2MG–LRP-1 pair has a great potential for the regulation of cytokine homeostasis in blood and tissue, a critical point in the pathogenesis of several diseases.

We have recently showed that α2MG is abundant in a specific subset of neutrophil-derived vesicles (called microparticles) [8], and to be a major determinant for their protective effects in experimental sepsis [9]. Soluble α2MG has a short systemic half-life in mice (~ 4 min) [10] being mainly cleared by the liver [11]. To maximize α2MG protective activity and study these effects in the absence of other proteins present in the natural vesicles, we established if synthetic microcapsules could recapitulate the biological functions of α2MG. Biodegradable microcapsules were generated with a layer-by-layer microencapsulation technique and loaded with α2MG. In a model of peritoneal sepsis the synthetic α2MG-microcapsules controlled bacterial load, leading to animal survival [9]. These initial experiments provided important proof-of-concept that manufacturing microcapsules enriched with α2MG was a viable strategy to replicate the bioactions of α2MG when present in natural microvesicles. However, little is known about the interaction and properties of the synthetic microcapsules with human primary cells. Herein, we focused on the physical and chemical characteristics of these new biodegradable microcapsules loaded with α2MG and have investigated their interaction and biological functions in human cells and experimental settings, revealing, for the first time, their translational potential for therapeutic approaches.

## 2 Materials and methods

Please refer to the Supplementary Material for details on protocols, materials and sources.

### 2.1 α2MG enriched-microcapsule generation

**Fig. 1:**
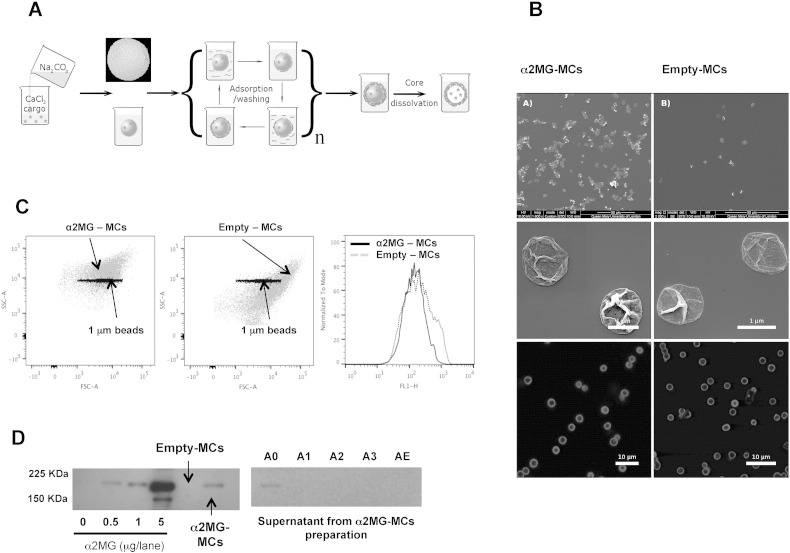
MC preparation and characterization. (A) MCs were generated using the layer-by-layer assembly protocol by alternate deposition of oppositely charged polyelectrolytes on sacrificial calcium carbonate template particles. (B) Morphology of α2MG-MCs (right panel) and empty-MCs (left panel) as shown by scanning electron and confocal microscopes. (C) Flow-cytometer analysis; forward and side scatter plots (left and middle panels): MCs (gray cloud); 1 μm beads (black cloud). Histograms (right panel): green fluorescence associated with the microcapsules. (D) Western Blot analysis of α2MG content in MCs in comparison with soluble protein.

Microcapsules (MCs) were prepared according to the LbL assembly technique by alternate deposition of oppositely charged polyelectrolytes on sacrificial calcium carbonate template microparticles (see Fig. 1 for schematic) [4]. α2MG was incorporated into the cores by co-precipitation at the particle synthesis stage, as described [9]. As a control, an empty preparation of MCs was used. Positively charged PLA and negatively charged DS were used for shell assembly and adsorbed from 2 mg/ml solutions in 0.15 M NaCl. One middle layer of FITC–PLL was adsorbed instead of PLA, used for the rest of positively charged layers, to fluorescently label microcapsules for confocal visualization and flow cytometry measurements. The final shell structure obtained was PLA/DS/FITC–PLL/[DS/PLA]_2_ with a positively charged outermost layer of PLA. After the shells were fully constructed, CaCO_3_ cores were dissolved in 0.2 M EDTA (pH 6.5). To estimate the encapsulation efficiency, supernatants were collected from particle synthesis, from the first three layer depositions and particle dissolution steps (named A0, A1, A2, A3, AE).

### 2.2 α2MG enriched-microcapsule characterization

Microcapsule morphology was characterized using FEI Inspect F scanning electron and Leica TS confocal microscopes. MCs were counted (obtaining values of 425 × 10^6^ and 264 × 10^6^ capsules/ml for α2MG- and empty-MCs, respectively) and analyzed by flow cytometry with BD LSRFortessa, together with 1 μm beads for comparison. The content of α2MG was assessed by Western blot analysis in α2MG-MCs, empty-MCs and supernatants from preparation steps (A0, A1, A2, A3, AE), loading soluble α2MG for comparison. To assess the efficiency of encapsulation, un-loaded protein was quantified by inverted ELISA. Standards (0.005–5 μg/ml of active α2MG) and supernatants A0, A1, A2, A3 and AE were i) incubated overnight at 4 °C; ii) an anti-α2MG (1:50,000; clone 2-M1 IIE7; BioMac) was applied for 2 h RT; iii) after washing and incubation with anti‐mouse HRP-conjugated antibody (1:5000; Invitrogen) for 2 h, 3,3′,5,5′-Tetramethylbenzidine (TMB) substrate buffer (R&D System) was added for 30 min; iv) the reaction was stopped with 1 N sulfuric acid (Sigma) and v) absorbance was read at 450 nm with a fluorescence plate reader.

### 2.3 In vitro biological analyses

#### 2.3.1 Preparation of human peripheral monocytes, monocyte-derived macrophages (MDM) and neutrophils

Peripheral blood neutrophils and monocytes were freshly isolated as described [12]. Purified monocyte population was obtained by adhesion (1 h, 37 °C, 5% CO_2_) and monocyte-derived macrophages (MDM) were prepared from monocytes, by culture (8–10 days) in RPMI 1640 containing 20% fetal bovine serum (FBS), glutamine and antibiotics [12].

#### 2.3.2 Flow chamber assay

To assess leucocyte–endothelial interaction, primary human umbilical vein endothelial cells (HUVEC) were collected and plated overnight in μ-Slides VI^0.4^ (Ibidi™) [13], [14]. The confluent monolayers were stimulated with TNF-α (10 ng/ml) in complete medium (M199) 0% FBS (to avoid contamination of exogenous α2MG), in the presence or absence of different amounts of MCs. Neutrophils were incubated for 10 min at 37 °C, and then perfused over endothelial cells at 1 dyn/cm^2^ for 8 min [14]. In another set of flow experiments, exogenous active α2MG was applied (9.4 ng/slide).

#### 2.3.3 Confocal microscopy

To visualize MCs and endothelial cell interaction, HUVEC and flown neutrophils were stained with Alexa Fluor® 546-Phalloidin (5 U/ml, Invitrogen) and left in Probing Antifade medium (Invitrogen) containing DAPI. They were visualized using a Zeiss LSM 510 META scanning confocal microscope and analyzed by Zeiss LSM Imaging software (Carl Zeiss). In another set of experiments, cells were stained with Alexa Fluor® 633-Wheat germ agglutinin (1 μg/ml; Invitrogen) followed by anti-active α2MG antibody (10 μg/ml, clone 2-M1 IIE7, BioMac), Alexa Fluor® 594 secondary antibody (Invitrogen) and Probing Antifade medium (Invitrogen) containing DAPI. By acquiring Z-stack images, the number of α2MG-positive particles on the membrane surface was acquired and counted in each sample using NIH ImageJ 1.48 software.

#### 2.3.4 Flow cytometry

Monocytes and MDM were assessed for both surface and intracellular expression of α2MG receptor (LRP1 or CD91, 5 μg/ml, clone A2Mr alpha-2, AbDSerotec) along with the lineage specific lineage marker: CD14 (0.5 μg/ml, clone 61D3, eBioscence) for monocytes and CD68 (0.5 μg/ml, clone Y1/82A, eBioscence) for MDM. Cells were then analyzed with a FACSCalibur flow cytometer using CellQuest™ and FlowJo software.

#### 2.3.5 Phagocytosis assay

MDM were evaluated for their ability to phagocytose Zymosan and *Escherichia coli* (*E. coli*) particles. MDM were incubated with different amounts of α2MG- or empty-MCs for 24 h (at 37 °C, 5% CO_2_). Zymosan (Zymosan A from *Saccharomyces cerevisiae*) and *E. coli* particles (Strain K12) were conjugated with a fluorescent dye (Bodipy® 576/589, 1 μM; Invitrogen). After 24 h of incubation with MCs, 125 μg/ml of fluorescent Zymosan particles or 1 mg/ml of fluorescent *E. coli* particles was added to the medium for a further 20 min or 1 h, respectively (at 37 °C, 5% CO_2_). The number of fluorescent phagocytized particles was determined with a fluorescence plate reader (BMG Labtech) and analyzed using MARS Data Analysis Software. Cells were further analyzed by a scanning confocal microscope. To further corroborate our phagocytosis results and discriminate between ingested and membrane-bound particles, human macrophages were incubated with microcapsules (1 × 10^5^/well) or soluble α2MG (94 ng/well) as described above and then incubated with phRodo *E. coli* (1 mg/ml, Invitrogen) for 30 min (37 °C, 5% CO_2_), following the manufacturer's instructions. The fluorescent emission of internalized particles was analyzed by flow cytometry (FACSCalibur using CellQuest™ and FlowJo software).

In another set of experiments Bodipy®-*E. coli* particle phagocytosis was monitored in biogel-elicited mouse macrophages following the same protocol above.

### 2.4 In vivo studies

C57Bl/6 mice (male, 6–8 weeks; Charles River) were used.

#### 2.4.1 Acute peritonitis

Vehicle (PBS), empty MCs (1 × 10^5^/mouse), α2MG-MCs (1 × 10^5^/mouse) or equivalent levels of soluble active α2MG (94 ng/mouse) were administered i.v. followed by i.p. administration of Zymosan A (0.1 mg). Peritoneal lavages were collected after 4 h and leukocyte infiltration was assessed by light microscopy, followed by differential analysis using anti-Gr-1 and anti-F4/80 staining and flow cytometry analysis.

#### 2.4.2 In vivo phagocytosis

Mice were injected with 1 ml of 2% Bio-Gel (Bio-Rad) i.p. and 3 days later, vehicle (PBS), empty MCs (1 × 10^6^/mouse), α2MG-MCs (1 × 10^6^/mouse) or soluble α2MG (940 ng/mouse) was administered i.p. After 18 h, mice were injected with fluorescent- (Bodipy® 576/589, 1 μM; Invitrogen) Zymosan A (1.6 mg i.p.) and peritoneal lavages were collected after 30 min. The fluorescence of engulfed particles in macrophages was evaluated by flow cytometry.

#### 2.4.3 Bioactive lipid quantification

Quantification of Protectin DX (PDX), Leukotriene B_4_ (LTB_4_), Prostaglandin E_2_ (PGE_2_), 5-Hydroxy Eicosatetraenoic acid (5-HETE), 15-Hydroxy Eicosatetraenoic acid (15-HETE), 14-Hydroxy Docosahexaenoic Acid (14-HDoHE), 17-Hydroxy Docosahexaenoic Acid (17-HDoHE), and 18-Hydroxy Eicosapentaenoic acid (18-HEPE) in peritoneal lavages, after phagocytosis assay, was achieved by LC–MS/MS measurements as described [15]. For each standard, calibration curves were built using 10 solutions at concentration ranging from 0.95 ng/ml to 500 ng/ml.

### 2.5 Statistical analysis

All statistical analyses were performed using GraphPad Prism (v6.0, San Diego CA, USA). Data are expressed as mean ± SEM of “*n*” independent experiments. Statistical evaluation was performed by One-way ANOVA with the Bonferroni post-test or unpaired Student's *t*-test when appropriated. Differences were considered statistically significant when p < 0.05.

## 3 Results

### 3.1 α2MG enriched-microcapsules and empty-microcapsules

Microcapsules were initially evaluated for their physicochemical characteristics. Morphological analyses using scanning electron (Fig. 1B upper panels) and confocal microscopes (Fig. 1B lower panels) indicated typical spherical nature of the microstructures with some folds likely created while drying. The sizes of individual capsules varied, as expected [4]. Some aggregation of microcapsules could be observed though not very pronounced. Comparing images from empty-MC and α2MG-MC, we concluded that encapsulation did not cause any noticeable morphological changes. Out of 6 distinct MC preparations using 0.5 or 1 mg α2MG for incorporation, a range of 160–425 × 10^6^/ml and 264–375 × 10^6^/ml α2MG-MCs and empty-MCs were produced (2 ml of total solution for each preparation). Flow cytometry was performed in relation to 1 μm beads, observing a diameter of ~ 1–2 μm (Fig. 1C), with no specific difference in fluorescence intensity between empty and α2MG-MCs (same units of fluorescence; Fig. 1C right panel).

To have a semi-quantitative and qualitative indication of α2MG incorporation in the capsules, Western blotting analysis was conducted. Fig. 1D illustrates an exemplar one with different capsule loadings.

In general, 1 × 10^6^ α2MG-MCs contained approximately 1 μg of α2MG. Furthermore, the majority of α2MG was lost in the initial step of incorporation as evident from detection in the A0 supernatant (Fig. 1D; right blot). Quantitative data were obtained by ELISA: congruently with the Western blotting data, only the A0 supernatant samples contained α2MG. We could calculate approximately 6.4 μg of unloaded protein, which is a minimal portion of the total amount of protein used for encapsulation (800 μg), yielding a calculated encapsulation of 94 ng of α2MG for 100,000 capsules. These microcapsules were tested in two systems where natural vesicles enriched with α2MG displayed bioactivity [9].

### 3.2 Biological effects of microcapsules

**Fig. 2:**
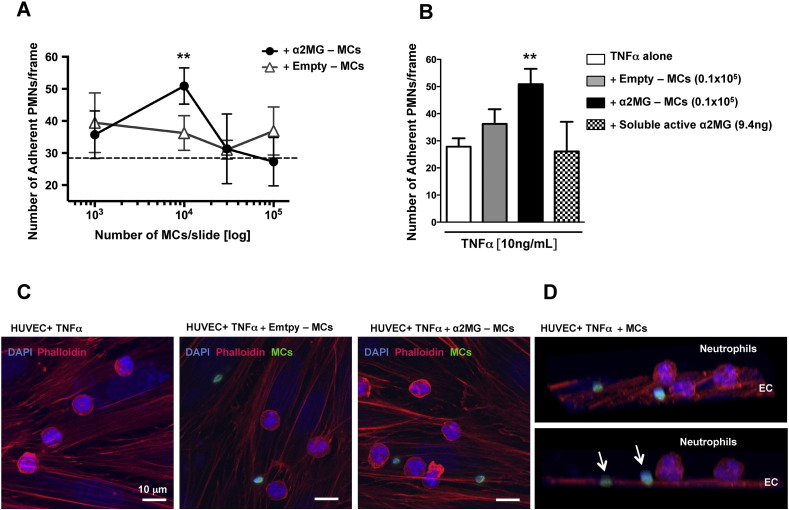
MC interaction with human endothelial monolayer. (A) Endothelial cells were treated with TNF-α (10 ng/ml; 4 h) in the presence or absence of different amounts of MCs (1 × 10^3^–1 × 10^5^) before neutrophils flow (see Materials and methods for protocol details). (B) MCs were compared to an equivalent amount of soluble α2MG (9.4 ng). Data are mean ± SEM of 9 different human donors for MC experiments, 3 for soluble α2MG (One way ANOVA, Bonferroni post-test, **p < 0.01 *vs.* vehicle). (C) Confocal images of endothelial monolayer treated with TNF-α and then vehicle (left panel), empty-MCs (central panel) and α2MG-MCs (right panel). Images show cells stained with Alexa Fluor® 546-Phalloidin (red) and DAPI (blue). Microcapsules are in green. (D) 3D images of α2MG-MC slide to demonstrate capsule insertion onto the endothelial monolayer (arrows). Images are representative of three distinct analyses.

First we tested α2MG-microcapsules and empty-microcapsules in the flow chamber assay with human neutrophils and human umbilical vein endothelial cells to corroborate the hypothesis that α2MG entrapped in synthetic structure retained its ability to promote cell-to-cell interaction. Thus, different amounts of α2MG-microcapsules, or empty-microcapsules, were incubated with TNF-α-stimulated endothelial cells for 4 h. Following flow at 1 dyn/cm^2^ of freshly isolated human peripheral blood neutrophils, a good extent of white blood cell adhesion was quantified with a significant effect of the capsules at 0.1 × 10^5^ dose (51 ± 6, 36 ± 5 and 28 ± 3 adherent cells with 0.1 × 10^5^ α2MG-microcapsules, empty-microcapsules or vehicle, respectively; **p < 0.01). Fig. 2A illustrates the concentration–response experiments, whereas Fig. 2B presents cumulative data for α2MG-microcapsules, empty-microcapsules and soluble α2MG (added to an equivalent amount of 9.4 ng). Confocal analyses of slides at the end of the 8 min flow experiment displayed microcapsule interaction with the human cells (Fig. 2C). Image reconstruction in Fig. 2D shows the chiefly insertion of the microcapsules within endothelial cells (white arrows). Collectively these results demonstrate that encapsulation technology ameliorates the pharmacological profile of α2MG at least within these experimental settings.

**Fig. 3:**
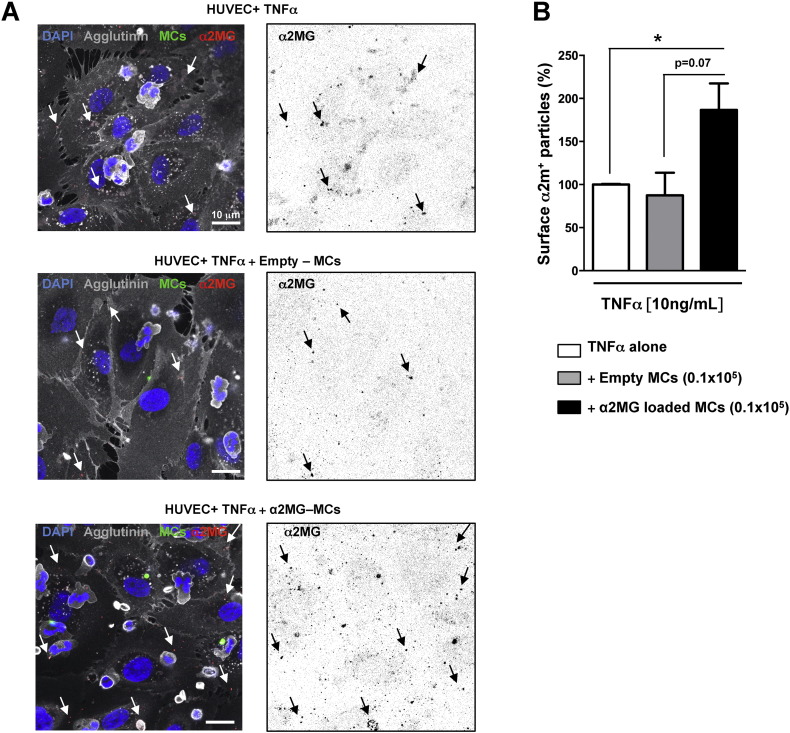
α2MG-microcapsules promote deposition of active α2MG on the surface of human endothelial cells. (A) Confocal images of endothelial monolayers treated with TNF-α as in Fig. 2, and then vehicle (upper panel), empty-MCs (central panel) or α2MG-MCs (bottom panel). Cells were stained with Alexa Fluor® 633-Wheat germ-agglutinin (gray), followed by anti-active α2MG antibody (red) and DAPI (blue). The intact microcapsules are in green. Z-stack images were acquired and the number of α2MG-positive particles on the endothelial membrane surface was counted in each sample (right panel for each group, black spots). Images are representative of three distinct analyses. (B) Cumulative data for α2MG-positive particles as determined by imaging software. Data are mean ± SEM of 3 independent experiments (Student's *t* test, *p < 0.05 *vs.* vehicle).

Next we wanted to visualize deposition of the protein on the human cells. To this aim, neutrophils and endothelial monolayers were stained with agglutinin along with a specific anti-α2MG antibody against the active conformation of the protein. Fig. 3A reports these images. Wheat germ-agglutinin recognizes sialic acid and N-acetylglucosaminyl residues on the plasma membrane, hence allowed us to analyze specifically the surface of endothelial cells; herein, a significant deposition of α2MG could be visualized (Fig. 3A). Two further notes are worthwhile. First, basal immunostaining for α2MG was evident in control settings and following incubation with empty microcapsules. Second, this was not particularly different between the two conditions whereas significant accumulation was quantified after α2MG-MCs (Fig. 3B). The incremented α2MG-immunostaining is not solely associated with the fluorescent capsules, indicating a potential release of the protein on the endothelial cell surface, possibly providing opportunity to interact with the recruited neutrophils.

**Supplementary Fig. 1:**
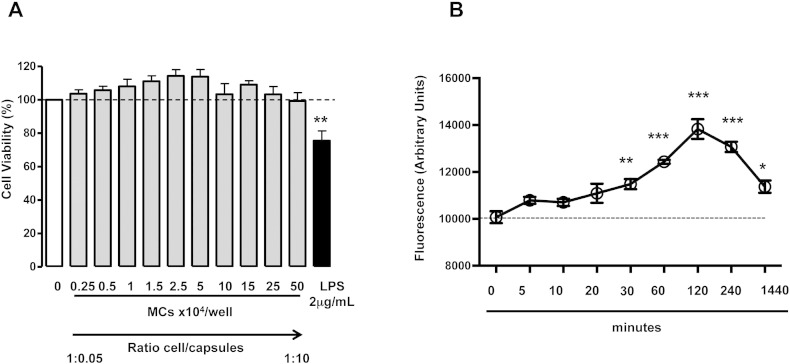
α2MG-microcapsules do not affect cell viability and show a time-dependent uptake in macrophages. (A) α2MG-MCs were added to RAW 246.7 cells, up to 10 capsules/cell for 24 h, along with a positive control (LPS, 2 μg/ml) and vehicle (PBS), prior to adding MTT solution. Formazan production was measured with a fluorescence plate reader. Data are mean ± SEM of 3 independent experiments (One way ANOVA, Bonferroni post-test, **p < 0.01 *vs.* vehicle). (B) Time-dependent uptake of MCs in macrophages, up to 24 h. Cell-associated fluorescence corresponding to the amount of phagocytosed capsules has being quantified with a plate reader. Data are mean ± SEM of 3 independent experiments (One way ANOVA, Bonferroni post-test, *p < 0.05, **p < 0.01, ***p < 0.001 *vs.* vehicle, dotted line).

**Supplementary Fig. 2:**
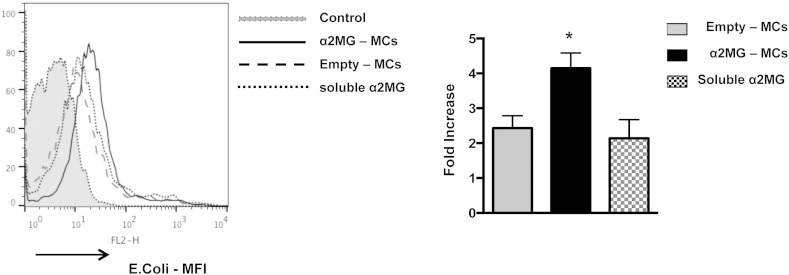
α2MG-microcapsules promote pH-sensitive-*E. coli* uptake in macrophages. Human macrophages were incubated with vehicle, α2MG-MCs, empty capsule (1 × 10^5^) or soluble α2MG (94 ng) for 24 h prior to application of pH-sensitive *E. coli* particles (1 mg/ml, 30 min). Data are mean ± SEM of 5 different human macrophage preparations, and are expressed as fold increase over control. Unpaired *t*-test, *p < 0.05 *vs.* α2MG-MCs.

**Supplementary Fig. 3:**
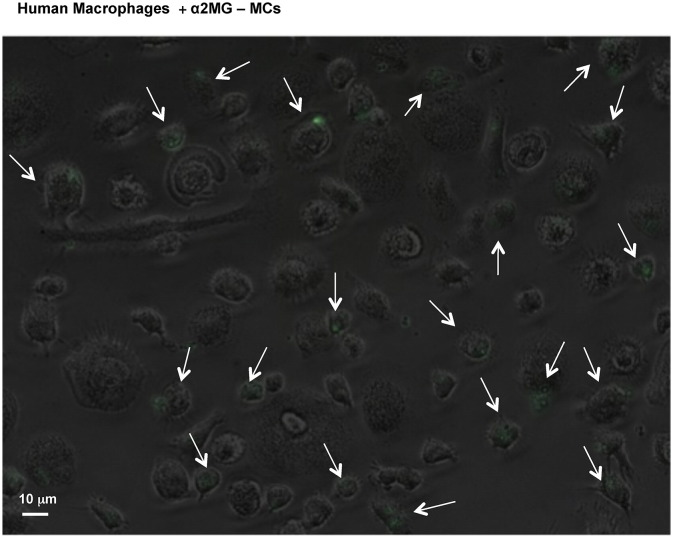
α2MG-microcapsule interaction with human monocyte-derived macrophages (MDM). Human macrophages were incubated with α2MG-MCs for 24 h prior to visualizing their update: a great disparity was observed across the cells. White arrows indicate cells that have engulfed a large number of capsules.

**Fig. 4:**
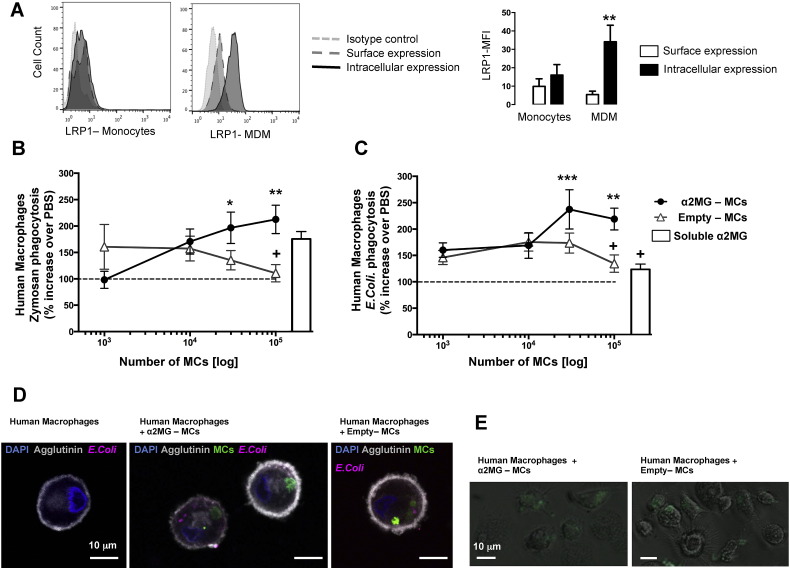
α2MG-MCs promote monocyte-derived macrophage (MDM) uptake of Zymosan and *E. coli* particles. Expression of LRP1 on human monocytes and MDM as quantified by flow cytometry in intact and permeabilized cells, along with CD14 for monocytes (0.5 μg/ml) and CD68 (0.5 μg/ml) for MDM. (A) Representative histograms showing augmented levels of LRP1 expression on MDM; bar graphs show cumulative data (mean ± SEM, n = 4; One way ANOVA, Bonferroni post-test, **p < 0.01 *vs.* MDM surface expression). (B) MDM uptake of fluorescent Zymosan (125 μg/ml, 20 min) or (C) *E. coli* (1 mg/ml, 1 h) particles upon MC application. Soluble α2MG (94 ng) was used for comparative purposes. Data are mean ± SEM of 4 different human donors. One way ANOVA, Bonferroni post-test, **p < 0.01 *vs.* vehicle, ^+^p < 0.05 *vs.* 1 × 10^5^ α2MG-MCs. (D) Representative confocal Z-stack images of MDM incubated with *E. coli* alone (vehicle) (left panel), α2MG-MCs and *E. coli* (central panel) or empty MCs and *E. coli* (right panel) as analyzed after phagocytosis. (E) Representative light microscopy images of MDM after 24 h incubation with MCs (1 × 10^5^ cells with 1 × 10^5^ MCs).

Next we determined if α2MG-MCs affected human macrophage phagocytosis. The putative α2MG receptor LRP1 is detected on human neutrophils [9], thus we determined its expression on human monocytes and monocyte-derived macrophages (MDM). LRP-1 (CD91) is generally recognized as a receptor critical for efferocytosis [16]. FACS analysis with an anti-CD91 antibody, along with the lineage specific markers CD14 for monocytes and CD68 for macrophages, revealed a predominant intracellular expression with augmented overall expression in the latter cell type (Fig. 4A). Thus, MDM were incubated for 24 h with α2MG-MCs or empty-MCs, prior to the addition of two distinct phagocytic stimuli. As depicted in Fig. 4B, incubation of MDM with α2M-MCs significantly promotes their ability to phagocytose Zymosan particles: this effect was concentration-dependent at 1 × 10^5^ and 0.3 × 10^5^ α2MG-MCs. A similar outcome was obtained with dead *E. coli* particles, though higher increments over soluble α2MG could be calculated (*e.g.* 220 ± 20%, 134 ± 16% and 139 ± 11% increased phagocytosis over vehicle for 1 × 10^5^ α2M-MCs, 1 × 10^5^ empty-MCs and 94 ng soluble α2MG, respectively; Fig. 4C). These effects were not due to alteration in cell viability as confirmed with human MDM (not shown) and RAW 246.7 cells (Supplementary Fig. 1A). Moreover, using pH sensitive *E. coli* particles we corroborated these results in human macrophages, demonstrating again a significantly increase of particle uptake in α2M-MC-treated cells as compared to empty capsules and soluble protein treatments (> 50%, *p < 0.05, Supplementary Fig. 2). Capsules entered into human MDM cell cytoplasm, as seen by confocal analysis, an effect independent from α2MG (Fig. 4D). Intact MCs could be detected even after the 24 h of incubation (Fig. 4D). Interestingly, light microscopy imaging indicated that the distribution of capsules was not homogenous among macrophages with evidence for a subset of cells that internalized one or more capsules, and others devoid of fluorescent material (Fig. 4E). This phenomenon is not novel (as seen in RAW 246.7 cells with polystyrene particles [17]) and it is more noticeable in low magnification (Supplementary Fig. 3).

### 3.3 α2MG-MCs have protective effects on a model of peritonitis

**Fig. 5:**
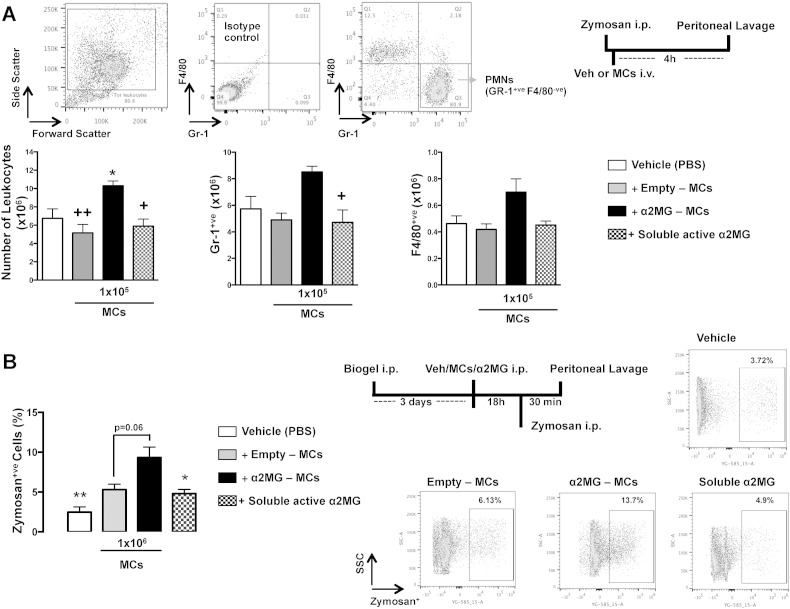
α2MG-MCs promote extravasation and phagocytosis within the peritoneal cavity. (A) Vehicle (PBS), empty MCs (1 × 10^5^/mouse), α2MG-MCs (1 × 10^5^/mouse) or α2MG (94 ng/mouse) was administered i.v. followed by Zymosan A (0.1 mg i.p.). Leukocyte infiltration was assessed in peritoneal lavages by differential flow cytometry using anti-Gr-1 and anti-F4/80. Example of gate strategy for Gr-1 +/F4 80 − neutrophils is showed in the upper panel. Data are M ± SEM of 5 mice/group (*p < 0.05 *vs.* vehicle, ^+^p < 0.05 *vs.* 1 × 10^5^ α2MG-MCs). (B) Vehicle, empty MCs and α2MG-MCs (1 × 10^6^/mouse), or soluble active α2MG (940 ng/mouse) were injected i.p. into mice which had received 3 days earlier an injection of biogel. After 18 h, fluorescent Zymosan A was injected and 30 min later the presence of engulfed particles in macrophages was evaluated by flow cytometry. Data report the percentage of macrophages positive for Zymosan particles (exemplar plots are on the right hand side). Data are mean ± SEM of 4 mice/group (unpaired *t*-test, *p < 0.05, **p < 0.01 *vs.* α2MG-MCs).

In the final part of the study we queried whether α2MG-MCs could be active during the complex and integrated settings of an inflammatory reaction. To parallel the *in vitro* data, we focused on neutrophil recruitment and macrophage phagocytosis. In a model of peritonitis, intravenous administration of α2MG-MCs (selected dose of 1 × 10^5^/mouse) augmented the extent of neutrophil recruitment to the site of inflammation: approximate + 60–80% increment in cell numbers over empty microcapsules or soluble α2MG (94 ng/mouse; Fig. 5A).

In line with the profiles of acute peritonitis [18], [19] neutrophil numbers and not macrophage levels were affected by the α2MG-MC treatment, as demonstrated by the use of cell-specific markers (Gr1 for granulocytes and F4/80 for macrophages) (Fig. 5A). Next, α2MG-MCs were tested for their ability to augment macrophage phagocytosis.

**Supplementary Fig. 4:**
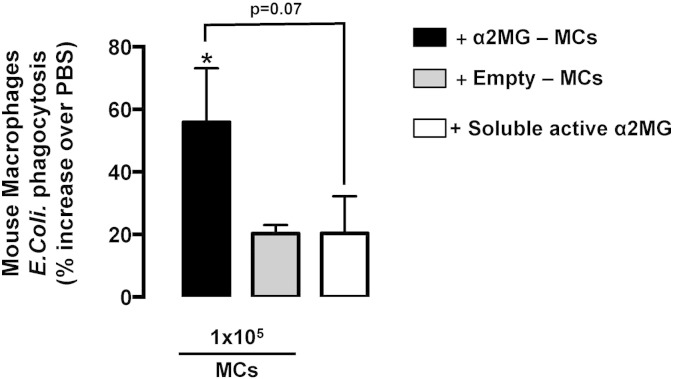
α2MG-microcapsules promote phagocytosis in biogel-elicited murine macrophages. Biogel-elicited mouse macrophages were incubated with vehicle (PBS), α2MG-MCs or empty MCs (1 × 10^5^/well) for 24 h before addition of fluorescent *E. coli* (1 mg/ml, 1 h) particles. Soluble α2MG (94 ng/well) was used for comparative purposes. The number of phagocytized particles was determined with a fluorescence plate reader. Data are mean ± SEM of 3 independent experiments (Student's *t* test, *p < 0.05 *vs.* vehicle).

Thus, macrophages were elicited into the peritoneal cavity prior to treatment i.p. with α2MG-microcapsules (1 × 10^6^/mouse), empty-microcapsules (1 × 10^6^/mouse), soluble α2MG (940 ng/mouse) or vehicle (PBS). The uptake of fluorescently labeled Zymosan A was assessed 18 h later, using a 30 min time point. Flow cytometry analysis allowed quantification of the cells engulfed with fluorescent particles, as presented in Fig. 5B. Mice treated with α2MG-MCs displayed an increased number of Zymosan^+ ve^ macrophages compared to the control group (> 70%, p < 0.01) and to soluble protein (> 50%, p < 0.05). These results were further corroborated in the *ex-vivo* setting with *E. coli* particles, highlighting a critical different pharmacology as compared with the soluble active protein (> 30%, Supplementary Fig. 4).

**Fig. 6:**
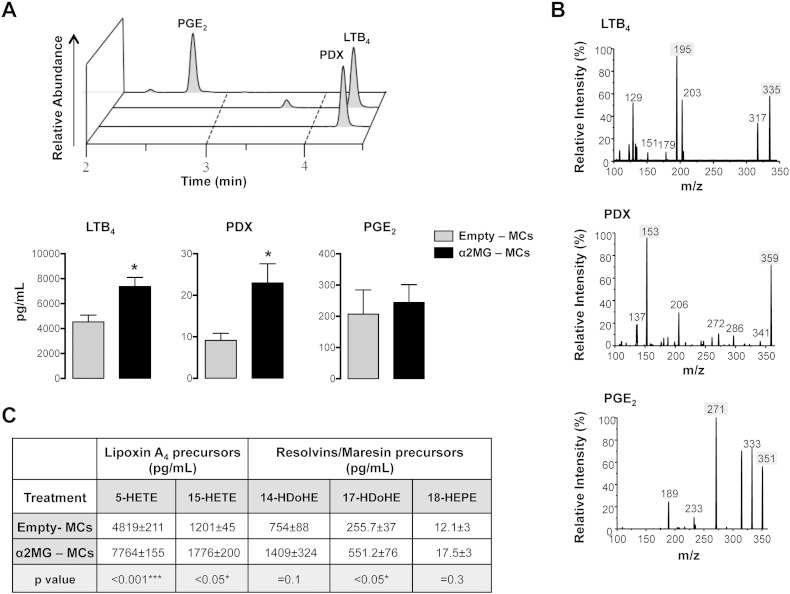
α2MG-MCs increase pro-resolving lipid mediators. Mice were subjected to the phagocytic assay as in Fig. 5B. Lipids of interest were determined in peritoneal lavage by LC–MS/MS. (A) Upper panel: Spectrum of relative peaks for Protectin DX, LTB_4_ and PGE_2_. Lower panel: LTB_4_, Protectin DX and PGE_2_ relative bar graphs. (B) Protectin DX, LTB_4_ and PGE_2_ signature fragment ions reported as m/z, highlighting the diagnostic fragments. (C) 5-HETE, 15-HETE, 14-HDoHE, 17-HDoHE, and 18-HEPE were identified and quantified by LC–MS/MS and divided according to their downstream lipid product. Data are mean ± SEM of 4 mice/group (Student's *t* test, *p < 0.05 *vs.* empty-MCs).

Finally to establish downstream mechanisms potentially responsible for the activating properties of α2MG-MCs on the acute inflammatory response, metabololipidomics was performed on cell-free peritoneal lavages. As depicted in Fig. 6, mice treated with α2MG-MCs, in comparison to empty-MCs, displayed higher levels of the pro-inflammatory chemoattractant LTB_4_, which derives from the lipoxygenation of arachidonic acid. The concentrations of the pro-resolving PDX were also augmented post-treatment with α2MG-MCs as compared to empty-MCs. Fig. 6A shows the chromatographic profiles for PDX, LTB_4_ and the cyclo-oxygenase product PGE_2_, and their cumulative values: it can be seen that the effects were not univocal and, for instance, levels of PGE_2_ were not altered by microcapsule administration. Fig. 6B shows the typical fragmentation patterns for these exemplar mediators following mass spectrometry. Moreover, the precursors for LXA_4_ and resolvins/maresins were augmented upon administration of the α2MG-MCs (Fig. 6C). Vehicle (PBS)-generated mediators were comparable to levels measured after treatment of mice with empty-MCs (not shown).

## 4 Discussion

We report here the generation of microcapsules loaded with α2MG and describe their biological efficacy on the modulation of human endothelial cell and macrophage reactivity. These analyses are completed with proof-of-concept experiments in the mouse that reveal the ability of α2MG – when delivered by the microcapsules – to incite the innate immune response, a mechanism that could underlie the reported efficacy in a model of sepsis [9]. Collectively these sets of experiments demonstrated that α2MG-MCs retain immune-modulatory properties during complex inflammatory settings, promoting neutrophil migration during acute inflammation and augmenting the phagocytic properties of macrophages.

Natural vesicles are emerging as a novel means for cell-to-cell communication with potential impact in physiology as well as pathology, which would include valid use as biomarkers [20] and indeed therapeutic tools [21]. However, these vesicles contain a large number of proteins and other cargoes, *e.g.* microRNA and lipids, which could limit their application or their efficacy over prolonged administration. For instance, an unwanted antibody reaction to specific proteins present in the vesicles would be detrimental for therapeutic development, even more so if the antigenic protein(s) result to be one that is not pivotal to the biological properties of interest. To this end, the exploding field of nanomedicines could come in succor allowing encapsulation of specific bioactive molecules and ameliorate their targeting or pharmacokinetics, in all cases impacting on the pharmacodynamics (*e.g.* see recent examples for an anti-inflammatory peptide [22], [23]). Herein we focused our attention onto α2MG since we demonstrated i) the presence of this protein in neutrophil-derived natural vesicles [8], ii) its segregated expression in vesicles quantified in patients surviving sepsis [9] and iii) its ability to modulate mouse cell reactivity, leading to partial protection in experimental sepsis [9].

Efficient encapsulation of α2MG into microcapsules was achieved, obtaining synthetic structures with the expected physical characteristics of shape, size and stability. This extends α2MG to the list of biologically active tools that can be entrapped in these dextran-based microstructures [5], [24]. In line with recent studies, the efficiency of encapsulation was high with a limited loss of protein during the process, calculated to be less than 1%. Encouraged by these results, then we tested α2MG-MCs in experimental conditions using human cells central to the immune response, these being endothelial cells and macrophages. Crucial comparisons were made with empty MCs, prepared alongside identical protocols, and/or soluble α2MG: our prediction was that α2MG-MCs could harness α2MG biology with higher efficacy over the soluble protein, presenting also specific biological properties when compared to the empty microcapsules. In human umbilical vein endothelial cells, α2MG-MCs led to increased adhesion of human neutrophils. This process is a hallmark of inflammation: neutrophils are the first cell type to reach the site of injury and in order to do so must initially interact with the vessel wall [25]. Using confocal studies, we could unveil an effective interaction and penetrance of the microcapsules into the endothelium with consequent exposure of active α2MG. A background level of α2MG was detected onto the monolayer, even in the absence of the addition of α2MG-MCs: this is plausibly due to the presence of the protein in the plasma added to the cell culture. In any case, we predicted that the active protein presented on the surface of endothelial cells would then interact with its receptor LRP1 presented by the rolling neutrophil, promoting the subsequent step of adhesion. LRP1 was initially described as a ‘silent’ endocytic receptor [26], but evidence is emerging that it can signal upon application of α2MG [27], [28]. The model that emerges is one where α2MG presented by the endothelial monolayer activated the flowing neutrophil promoting the transition from rolling to adhesion, thus committing to extravasation.

**Supplementary Fig. 5:**
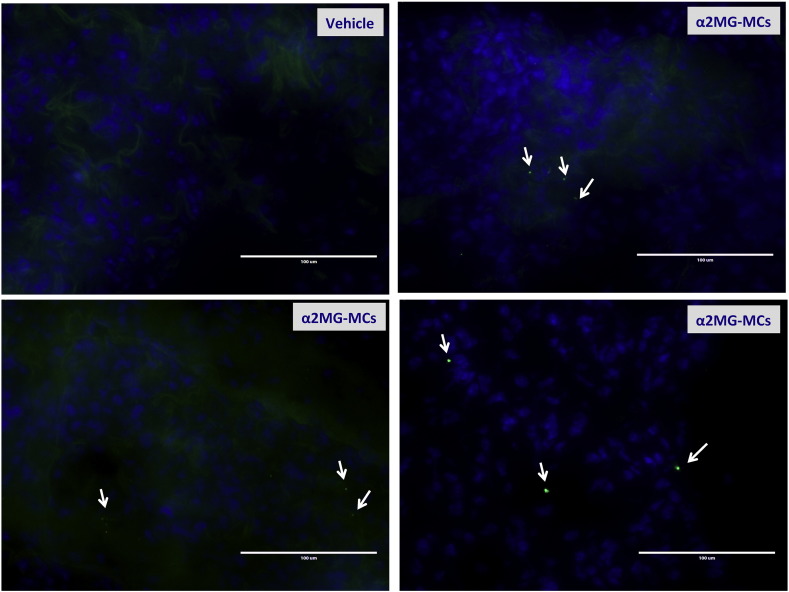
α2MG-microcapsules localize into the lung tissue once injected intravenously. Mice were treated i.v. with α2MG-MCs or PBS and organs collected 24 h later. MCs were visualized in the lung tissue sections selectively in mice treated with α2MG-MCs. Images are representative of three distinct analyses.

Meanwhile further studies are required to address the molecular events evoked by α2MG engagement of LRP1 on the rolling neutrophil, it is noteworthy that the model presented herein held consistency *in vivo*. Intravenous injection of α2MG-microcapsules – at doses coherent with what used *in vitro* when one considers an approximate total blood volume of 3–5 ml in the mouse – augmented neutrophil extravasation during on-going peritonitis. Such an effect would be beneficial on the way the host could combat an infective status, as discussed below. Intravenous injection of 1 × 10^5^ capsules confirmed their inability to cause toxicity (all animals survived). Furthermore, a specific experiment was performed to localize the injected capsules in the liver, spleen and lung. This was partially successful, since strong autofluorescence of the liver and spleen hampered easy detection of the capsules (data not shown). In contrast, we could identify α2MG microcapsules only in lung tissue sections by fluorescent microscopy (Supplementary Fig. 5). However, we are confident that the capsules would have been taken up by the liver and spleen, as demonstrated in a recent study using capsules with similar chemical composition [29]. Next, we focused our attention onto the human macrophages, central to initiation and resolution of the inflammatory response [30] and the main cell type devoted to phagocytosis.

Initial works with similar microcapsules have demonstrated uptake of the microstructures by immortalized macrophage cell lines, both from human and murine origins [31] however studies with primary cells are lacking. The addition of α2MG-microcapsules onto human monocyte-derived macrophages resulted in a time-dependent uptake (Supplementary Fig. 1B). At variance from the human endothelial cells, macrophage uptake was optimal over a longer time-point, with maximal result at 24 h incubation. However, this kinetics is similar to that observed for the empty microcapsules and it is, therefore, a feature of these microstructures and not secondary to the physical presence of α2MG or one of its biological effect. Interestingly, only a subset of macrophages was shown to take up the microcapsules. This was a consistent result with a proportion of cells engulfing well over one or two microcapsules and other adjacent cells being empty, with no microcapsules at all. The molecular explanation of this dichotomy of response is unclear however it is not uncommon and previously observed for other phagocytic stimuli [17].

Once inside the human macrophage, α2MG was in part released by the microcapsules and consistently promoted phagocytosis of other stimuli, as shown with two unrelated particles of pathogenic relevance, Zymosan A that is a major component of the yeast wall and the Gram negative bacterium *E. coli*. In line with the kinetics of the phagocytosis process (*e.g.* see our work with mouse macrophages and endogenous Annexin A1 [32]), the engulfment of these pathogenic particles was rapid with significant values within 30–45 min, yet augmented by previous uptake of the α2MG-MCs, as demonstrated by two experimental approaches using different preparations of *E. coli* (Fig. 4C and Supplementary Fig. 2). It is yet unknown how the α2MG-MCs would accelerate this response of the macrophage and this will require further studies. The cytoplasmic domain of LRP1 contains the necessary structural features to mediate phagocytosis [33]; it is unclear whether α2MG would interact with LRP1 onto the phago-lysosome, inside the macrophage, or if the protein is externalized to interact with LRP1 in a paracrine or juxtacrine fashion.

The experiments with human monocytes and MDM provided another interesting observation: LRP1 was present in the former though increased expression was quantified upon differentiation in the latter, reflecting the phagocytic role of macrophages [34]. We concluded this set of experiments by reasoning again that it was important to establish whether the positive modulation of macrophage phagocytosis could be replicated *in vivo*. To this end, intraperitoneal injection of α2MG-MCs promoted the uptake of fluorescent Zymosan and increased lipid mediators' levels. The metabololipidomics revealed interesting mechanistic connections. While the augmented level of LTB_4_ in α2MG-MC-treated mice is in accordance with the enhanced extent of neutrophil recruitment we showed in peritonitis, the elevation of PDX was of particular interest. This bioactive compound is produced *via* sequential lipoxygenation of docosahexaenoic acid (22:6 *n*-3) and can be found together with the first identified protectin, protectin D1 (PD1) in mouse inflammatory exudates [35]. Moreover, PDX and the other bioactive tissue-protective mediators including the LXA_4_ and resolvins or maresins are potent inducers of macrophage phagocytosis [36], [37]. It is of further interest that α2MG-MCs afforded significant modulation of lipoxygenase dependent metabolism with little or no detectable effect on the cyclo-oxygenase pathways.

Altogether these new results complement well the macroscopic amelioration of experimental sepsis [9], [38]. Our new data with human cells and the mechanism revealed herein indicate that α2MG-MCs could be developed for the management of human sepsis. From a mechanistic perspective, efficacy in septic settings would derive from an augmentation of neutrophil extravasation and macrophage phagocytosis, cellular event of paramount importance in the way the host would deal with the bacterial attack. A more general implication entails the appreciation that synthetic microcapsules, filled with key players of the inflammatory process, can harness their tissue-protective biology by prolonging stability in the organism, and ultimately represent a novel strategy for the delivery of therapeutics.

The following are the supplementary data related to this article.

Supplementary material.
