# Supplementary material for: Alpha-2-macroglobulin loaded microcapsules enhance human leukocyte functions and innate immune response
Source: J Control Release. 2015 Nov 10;217:284–92. doi: 10.1016/j.jconrel.2015.09.021 (PMC4649706; doi:10.1016/j.jconrel.2015.09.021)
Supplement: Supplementary file 1 — Supplementary material. [file mmc1.docx]

**SUPPLEMENTARY MATERIALS AND METHODS**

***Reagents***

Unless otherwise specified, materials were obtained from Sigma-Aldrich Ltd (Poole, UK). Poly-L-Arginine Hydrochloride (PLA, molecular weight 15-70 kDa), FITC-labeled Poly-L-lysine Hydrobromide (FITC-PLL, molecular weight 30-70 kDa), Dextran Sulfate sodium salt (DS, molecular weight ~100 kDa) were used as polyelectrolytes for microcapsules shells adsorption. Soluble active α2MG was obtained from BioMac (Leipzig, Germany) together with specific anti-α2MG antibody (clone α2-Mα1 IIE7). α2MG antibody clone 257316 was used for Western blotting analyses (R&D System, Abingdon, UK). Alexa Fluor® 594 secondary antibody and Alexa Fluor® 633-Wheat germ Agglutinin were from Invitrogen. Anti-CD14 antibody (clone 61D3) and anti-CD68 antibody (clone Y1/82A) were purchased from eBioscence (Hatfield, UK). Anti-LRP1 antibody (clone A2Mr alpha-2) was from AbDSerotec (Hatfield, UK).

***α2MG enriched-microcapsule generation***

Microcapsules were prepared according to LbL assembly technique by alternate deposition of oppositely charged polyelectrolytes on sacrificial calcium carbonate template microparticles (see Figure1 for schematic) [1]. α2MG was incorporated into the cores by co-precipitation at particles synthesis stage, similarly to how it was done previously [2]. Cargo molecules can be successfully trapped to CaCO_3_ particles provided by their porous inner structure. Sacrificial CaCO_3_ templates were synthesized immediately before microcapsules preparation by mixing of 0.33M CaCl_2_ and Na_2_CO_3_ solutions while vigorously stirring at magnetic stirrer, according to a well-established protocol [1]. For co-precipitation, 800µL of 1mg/ml α2MG solution in PBS was mixed with 1ml of CaCl_2_ prior to addition of 1ml of Na_2_CO_3_. As a control, an empty preparation of microcapsules was used, prepared and handled exactly as the α2MG-microcapsules but devoid of any protein. Shells were assembled on freshly prepared cores by alternate adsorption of oppositely charged polyelectrolytes with triple intermediate centrifugation/washing steps. Positively charged PLA and negatively charged DS were used for shell assembly and adsorbed from 2 mg/ml solutions in 0.15M NaCl. One middle layer of FITC-PLL was adsorbed instead of PLA, used for the rest of positively charged layers, to fluorescently label microcapsules for confocal visualization and flow cytometry measurements. The final shell structure obtained was PLA/DS/FITC-PLL/[DS/PLA]_2_ with positively charged outermost layer of PLA. After the shells were fully constructed, CaCO_3_ cores were dissolved in 0.2 M EDTA (pH 6.5) followed by triple washing and centrifugation steps first in EDTA and then in water.

To estimate the encapsulation efficiency, supernatants were collected from particles synthesis, from the first three layers depositions and particles dissolution steps (named A0, A1, A2, A3, AE). Together with these, supernatants from empty capsules preparation steps were collected. The amount of unloaded α2MG in the supernatants was then measured by inverted ELISA (see below).

***α2MG enriched-microcapsules characterization***

After construction, microcapsules morphology was characterized using FEI Inspect F scanning electron and Leica TS confocal microscopes. For SEM imaging, a drop of microcapsules dispersion was dried upon a glass slide fixed to sample holder, and then sputtered with gold. Imaging was performed at acceleration voltage of 10 kV. As can be seen from SEM and confocal images (Figure 1B) microcapsules of all types used for experiments have good circular shape, collapsing upon drying to a characteristic pancake-like structure. The sizes of individual capsules vary, which is a known feature of CaCO_3_-based microcapsule [1]. Some aggregation of microcapsules can be seen in images, but it is not very pronounced. Comparing the images between each other, it can be concluded, that encapsulation of α2MG does not lead to any noticeable structural or morphological changes of microcapsules.

α2MG-MCs and empty-MCs were then counted with a haemocytometer, obtaining values of 425x10^6^ and 264x10^6^ capsules/ml for α2MG-and empty preparation, respectively. The microstructures were then analyzed with BD LSRFortessa (BD Biosciences, San José, California) using forward and side scatter analysis, together with 1 μm beads for comparison (Invitrogen, Paisley, UK). No differences were quantified in the extent of FL-1 (FITC) fluorescence signals.

The content of α2MG was then assessed by Western Blot analysis: 20 μl of α2MG -microcapsules, empty-microcapsules solution and 5 preparations of the supernatants collected from preparation and washing steps (A0, A1, A2, A3, AE) were freeze/thawed (3 cycles), sonicated in water bath, boiled at 90 °C for 10 min before loading into a 8% acrylamide gel and electro-blotted on PDVF membrane. Different amounts of soluble α2MG were also loaded for comparison. Membrane was incubated overnight with specific α2MG antibody (1 μg/ml in 5% milk; clone 257316, R&D System) at 4 °C and then detected with anti-mouse HRP-conjugated antibody (Dako, Glostrup, Denmark). Chemi-luminescence signals were analyzed under non-saturating conditions with an image densitometer (FluorChem E, ProteinSimple, Santa Clara, USA). As can be seen in Figure 1D, unloaded protein was found in the first supernatant collected from the preparations steps.

To assess the efficiency of encapsulation, the amount of unloaded protein was quantified by inverted ELISA. Standards (0.005–5 μg/ml of active α2MG) and supernatants A0, A1, A2, A3 and AE were i) incubated overnight at 4°C; ii) an anti- α2MG (1:50.000; clone α2-Mα1 IIE7; BioMac, Leipzig, Germany) was applied for 2h RT; iii) after washing and incubation with anti‐mouse HRP-conjugated antibody (1:5000; Invitrogen) for 2h, 3,3′,5,5′-Tetramethylbenzidine (TMB) substrate buffer (R&D System) was added for 30 min; iv) the reaction was stopped with 1N sulphuric acid (Sigma) and v) absorbance read at 450 nm with a fluorescence plate reader (NOVOstar, BMG LABTECH, Aylesbury, UK) and analysed using MARS Data Analysis Software (version 1.20 R2, NOVOstar, BMG Labtech, Aylesbury, UK).

***In vitro biological analyses***

*Preparation of human peripheral monocytes, monocyte-derived macrophages (MDM) and neutrophils.* All volunteers gave written informed consent to blood collection and the procedure was approved by the East London & The City Local Research Ethics Committee (Rec Ref. 05/Q0603/34 ELCHA, London, United Kingdom) in accordance with the World Health Organization guidelines on drawing blood. Peripheral blood from healthy donors (50 ml) was collected by intravenous withdrawal and added to 3.2% w/v sodium citrate solution to prevent coagulation. Samples were processed by centrifuging at 150g for 20 min at RT. Platelet-rich plasma was removed and Dextran 6% w/v (Sigma) in Dulbecco phosphate-buffered saline (DPBS, Sigma) was gently added for 20 min. The leukocyte rich layer was carefully collected, layered over Histopaque, density 1.077 g/cm^3^ and centrifuged at 400 g for 30 min at room temperature. After gradient centrifugation, monocytes and neutrophils were both collected. The monocyte-enriched layer was recovered by thin suction at the interface, as described [3]. Cells were washed and re-suspended in RPMI 1640 medium supplemented with L-glutamine (2 mM), 50 μg/ml streptomycin and 5 U/ml penicillin. Purified monocyte populations were obtained by adhesion (1 h, 37 °C, 5% CO_2_), non-adherent cells (mainly lymphocytes) being gently removed with sterile DPBS. Monocyte-derived macrophages (MDM) were prepared from monocytes, by culture (8-10 days) in RPMI 1640 medium containing 20% fetal bovine serum (FBS), glutamine and antibiotics (as above). This method ensures the differentiation of monocytes towards macrophage-like cells as previously described [3]. Immediately after removing the monocyte-enriched layer, the lower layer containing neutrophils was also collected and the contaminating few erythrocytes were removed by hypotonic lysis.

*Flow chamber assay.* Umbilical cords were supplied by the midwifery staff of the maternity unit Royal London Hospital, and processes as approved by the East London & The City Local Research Ethics Committee (Rec Ref. 05/Q0603/34 ELCHA, London, United Kingdom). Primary human umbilical vein endothelial cells (HUVEC) were isolated by collagenase digestion of the interior of the umbilical vein [4] and collected in T75 flasks in complete medium (M199) (Sigma) supplemented with penicillin (100U), streptomycin (100 mg/ml), amphotericin B (2.5 μg/ml), L-glutamine (2 mM) and 20% of human serum (HS, Lonza, Cambridge, UK). To assess leucocyte-endothelial interaction, HUVEC were plated overnight in µ-Slides VI^0.4^ (Ibidi™, Munchen, Germany). In order to avoid contamination of exogenous α2MG present in the serum, before each experiment, the medium was replace with complete medium (M199) 0% serum. The confluent monolayers were then stimulated with TNF-α (10 ng/ml, Sigma) in complete medium (M199) 0% serum, in presence or absence of different amounts of α2MG-MCs or empty-MCs for 4h, at 37°C. Immediately prior to flow, freshly prepared neutrophils were suspended at 1x10^6^/ml in DPBS supplemented with Ca^2+^ and Mg^2+^ containing 0.1% bovine serum albumin (BSA) and incubated for 10 min at 37 ˚C. The entire flow chamber was placed under a Nikon Eclipse TE3000 microscope fitted with a x20 phase contrast objective (Nikon). Neutrophils were perfused over the monolayer at 1 dyne/cm^2^ using a programmable syringe pump (Stoelting, Dublin, Ireland) for 8 min, and then 6 random fields/treatment were recorded for 10 seconds each. Sequences were loaded into ImagePro-Plus software (Media Cybernetics, Wokingham, UK), neutrophils were tagged and their migration monitored. The total number of interacting neutrophils was quantified as captured and further classified as rolling or adherent if stationary for the 10 sec period [5]. In another set of flow experiments, exogenous α2MG in its active conformation (BioMac, Germany) was employed to compare the effects of soluble protein towards microcapsules-entrapping protein (9.4 ng/slide, being the corresponding amount of protein to the effective dose of 10.000 capsules).

*Confocal microscopy.* To visualize microcapsules and endothelial cell interaction, after the flow assay, HUVEC and flown neutrophils were immediately fixed with 1% paraformaldehyde (PFA) for 10 min at 4˚C and then washed with PBS. Cells were stained with Alexa Fluor® 546-Phalloidin (5 U/ml, Invitrogen) for 20 min RT in the dark. Cells were then washed in PBS and left in Probing Antifade medium (Invitrogen) containing DAPI. They were visualized using a Zeiss LSM 510 META scanning confocal microscope (x63 oil-immersion objectives) and analyzed by Zeiss LSM Imaging software (Carl Zeiss, Oberkochen, Germany). In another set of experiments, cells were stained with Alexa Fluor® 633-Wheat germ Agglutinin (1 μg/ml; Invitrogen) for 20min RT in the dark and then blocked for 30min in PBS containing 5% FBS. Unconjugated primary anti-α2MG antibody specifically raised against active α2MG (10 μg/ml, clone α2-Mα1 IIE7, BioMac) was then added for 1 h in PBS 1% FBS, followed by Alexa Fluor® 594 secondary antibody (Invitrogen) and Probing Antifade medium (Invitrogen) containing DAPI before analyzing as described before. Moreover, by acquiring Z-stack images, the number of α2MG-positive particles on the membrane surface were acquired and counted in each sample using NIH ImageJ 1.48 software (NIH, Bethesda, USA).

*Flow cytometry.* Monocytes and monocyte-derived macrophages (MDM) were assessed for both surface and intracellular expression of α2MG receptor (LRP1 or CD91) along with the lineage specific lineage marker (CD14 for monocytes, CD68 for MDM). Cells were washed with ice-cold PBS and scraped in ice-cold PBS containing 1% FBS. To analyze the intracellular expression cells were incubated with permeabilization buffer containing 0.1% saponin (eBioscence, Hatfield, UK) following the manufacturer’s instructions. Both un-permealized and permealized cells were labeled with anti-CD14 antibody for monocytes (0.5 μg/ml; clone 61D3, eBioscence) and anti-CD68 antibody (0.5 μg/ml; clone Y1/82A, eBioscence) for MDM together with anti-CD91 FITC-conjugated antibody (5μg/ml; clone A2Mr alpha-2, AbDSerotec) antibodies and with the relevant isotype controls, for 1 h at 4 ˚C in the dark. Cells were then washed and analyzed with a FACSCalibur flow cytometer (Becton Dickinson, San Jose, CA) using CellQuest TM software (Becton Dickinson) and FlowJo software (OR, USA).

*Phagocytosis assay.* MDM were evaluated for their ability to phagocytose Zymosan and *Escherichia Coli* (*E. Coli*) particles. MDM were seeded in 96-well black plates at a density of 1x10^5^ cells per well. Cells were first washed in sterile DPBS and then incubated in RPMI containing 0.1% FBS with different amounts of α2MG-MCs and empty MCs for 24 h (at 37 °C, 5% CO_2_). Zymosan (Zymosan A from *Saccharomyces Cerevisiae*, Sigma) and *E. Coli* particles (Strain K12, Sigma) were conjugated with a fluorescent dye (Bodipy® 576/589, 1 μM final concentration; Invitrogen) and un-labeled particles washed by centrifugation in PBS at 400 g for 10 min. After 24 h of incubation with microcapsules, Zymosan fluorescent-labeled particles were added to the medium at a final concentration of 125 µg/ml and cells were incubated for a further 20 min (at 37 °C, 5% CO_2_). In another set of experiments, *E. Coli* fluorescent-particles were added at a final concentration of 1 mg/ml and MDM were incubated for a further 60 min (at 37 °C, 5% CO_2_). Cells were gently washed 4 times with ice-cold PBS to remove extracellular particles. The number of fluorescent phagocytized particles was determined with a fluorescence plate reader (BMG Labtech) and analysed using MARS Data Analysis Software (version 1.20 R2, BMG Labtech). To further corroborate our phagocytosis results and discriminate between ingested and membrane-bound particles, human macrophages were incubated with microcapsules (1x10^5^/well) or soluble α2MG (94ng/well) as described above and then incubated with phRodo *E. Coli* (1mg/ml, Invitrogen) for 30 min (37°C, 5% CO_2_), following manufacture’s instructions. The fluorescent emission of internalized particles was analyzed by Flow cytometry (FACSCalibur using CellQuest TM and FlowJo software).

In another set of experiments Bodipy®-*E.Coli* particle phagocytosis was monitored in biogel-elicited mouse macrophages following the same protocol described above.

*Microcapsule toxicity and uptake assays*. Cell viability in presence of microcapsules was tested using MTT (3-[4,5-dimethylthiazol-2-yl]-2,5 diphenyl tetrazolium bromide) assay. Briefly, RAW 264.7 cells were cultured in Dulbecco's Modified Eagle's Medium *(*DMEM)*,* L-glutamine (2 mM), 50 μg/ml streptomycin, 5 U/ml penicillin and 10% FCS and incubated with different amount of microcapsules (0.25-50x10^4^/0.5x10^5^ cells) along with a positive control (LPS 2 μg/ml) and vehicle (PBS) for 24 hours. MTT (0.5 mg/ml, Sigma) was added to the medium for further 2 hours and DMSO was used to solubilize the formazan produced by metabolically active cells. The corresponding absorbance signal at 570 nm was measured with a fluorescence plate reader (BMG Labtech).

Macrophage uptake of capsules was also monitored over a time-course up to 24 hours post-application. As before, RAW 264.7 cells were plated with 1x10^5^ capsules and, for each time point, washed with PBS to removed non-phagocytosed capsules, with cell-associated fluorescence being quantified with a plate reader (NOVOstar, BMG LABTECH) at excitation and emission wavelengths of 485 and 520nm, respectively.

***In Vivo studies***

*Acute Peritonitis.* All animal studies were approved and performed under the guidelines of the Ethical Committee for the Use of Animals, Barts and The London School of Medicine and Home Office Regulations (Guidance on the Operation of Animals, Scientific Procedures Act, 1986). Peritonitis and *in vivo* phagocytosis were assessed in C57Bl/6 mice (male, 6-8 weeks) purchased from Charles River (Kent, UK). In the peritonitis experiments, vehicle (PBS), empty MCs (1x10^5^/mouse), α2MG-MCs(1x10^5^/mouse) or equivalent levels of soluble active α2MG (94 ng/mouse) were administered i.v. followed by i.p. administration of Zymosan A (0.1 mg; Sigma-Aldrich). Peritoneal lavages were collected after 4h and leukocyte infiltration was assessed by light microscopy, followed by differential analysis using anti-Ly6G (Gr-1) (clone RB6-8C5, eBioscence) and anti-F4/80 (clone BM8, BD Pharmingen) staining and flow cytometry analysis.

*In vivo* *phagocytosis.* Mice were injected with 1 ml of 2% Bio-Gel (Bio-Rad) i.p. and 3 days later, vehicle (PBS), empty MCs (1x10^6^/mouse), α2MG-MCs (1x10^6^/mouse) or soluble α2MG (940 ng/mouse) were administered i.p. for further 18 h. Mice were then injected i.p. with fluorescent- (Bodipy® 576/589, 1 μM; Invitrogen) Zymosan A (1.6 mg; Sigma-Aldrich) and peritoneal lavages were collected after 30 minutes. The fluorescence of engulfed particles in macrophages was evaluated by flow cytometry analysis (Becton Dickinson, San Jose, CA) using CellQuest TM software (Becton Dickinson) and analyzed with FlowJo software (OR, USA).

*Bioactive lipid quantification.* Quantification of Protectin DX (PDX), Leukotriene B_4_ (LTB_4_), Prostaglandin E_2_ (PGE_2_), 5-Hydroxyeicosatetraenoic acid (5-HETE), 15-Hydroxyeicosatetraenoic acid (15-HETE), 14-Hydroxy Docosahexaenoic Acid (14-HDoHE), 17-Hydroxy Docosahexaenoic Acid (17-HDoHE), 18-Hydroxy Eicosapentaenoic acid (18-HEPE) in peritoneal lavages, after phagocytosis assay, was achieved by LC–MS/MS measurements as described [6]. Briefly, to simultaneously separate endogenous lipids of interest, and 3 deuterated internal standards, LC-MS/MS analysis was performed on UHPLC system (Agilent LC1290 Infinity) coupled to Agilent 6460 triple quadruple MS (Agilent Technologies, Les Ulis, France) equipped with electro-spray ionization operating in negative mode. Reverse-phase UHPLC was performed using ZorBAX SB-C18 column (2.1 mm, 50 mm, 1.8 µm) (Agilent Technologies) with a gradient elution. Data were acquired in Multiple Reaction Monitoring (MRM) mode with optimized conditions (ion optics and collision energy). Peak detection, integration and quantitative analysis were done using Mass Hunter Quantitative analysis software (Agilent Technologies). For each standard, calibration curves were built using 10 solutions at concentration ranging from 0.95 ng/ml to 500 ng/ml.

*In vivo microcapsules localization.* Mice were injected with α2MG-MCs (1x10^5^/mouse) or PBS intravenously and 24h later liver, spleen and lungs have been collected, embedded in a block of cryopreservation medium (OCT) and sectioned for fluorescent microscopy examination. Images have been analyzed with EVOS® FL Cell Imaging System (Thermo Scientific, Loughborough, UK).

***Statistical analysis***

All statistical analyses were performed using GraphPad Prism (v6.0, San Diego CA, USA). Data are expressed as mean ± SEM of “*n*” independent experiments. Statistical evaluation was performed by One-way ANOVA with Bonferroni post-test or unpaired Student’s t-test when appropriated. Differences were considered statistically significant when *p* < 0.05.

**SUPPLEMENTARY FIGURES**

**
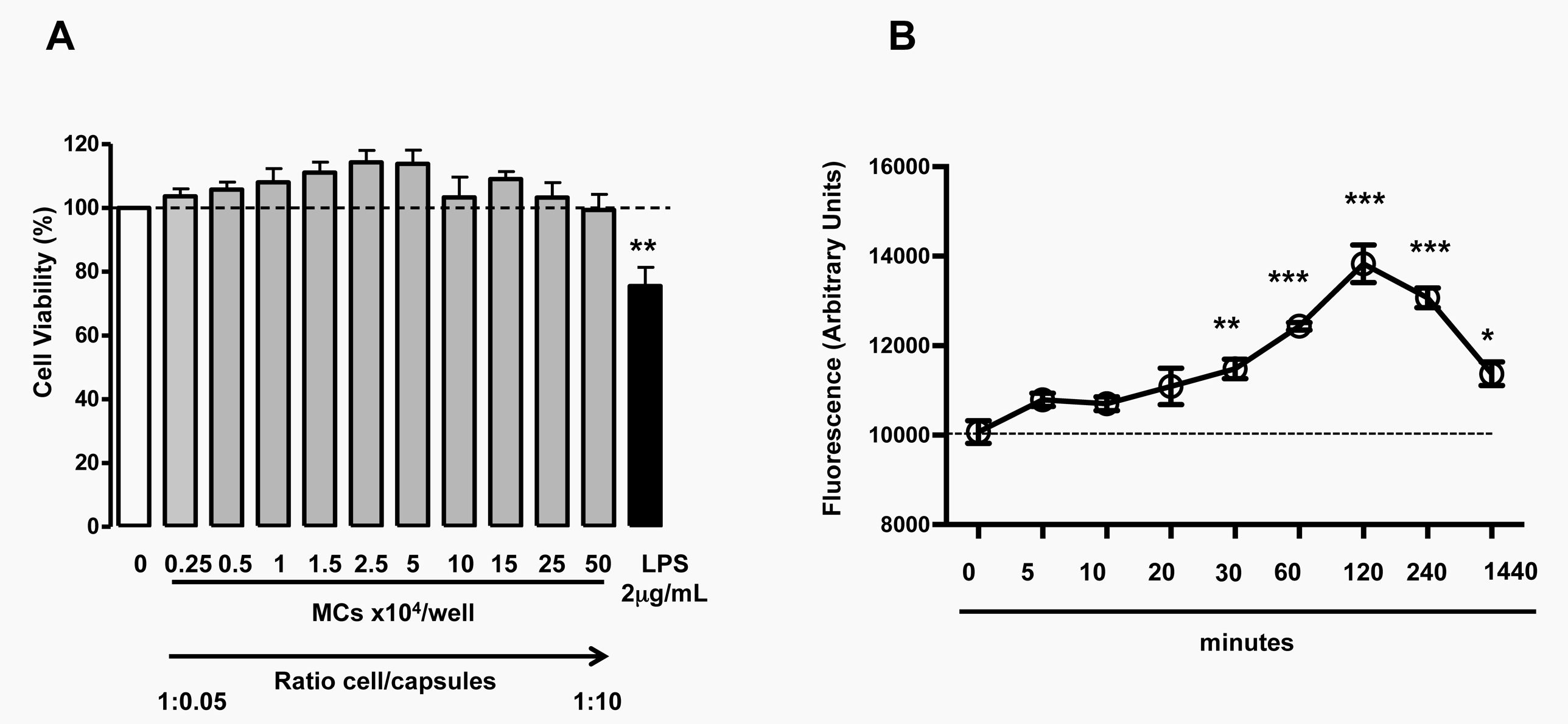
**

**Supplementary Figure 1. α2MG-microcapsules do not affect cell viability and show a time-dependent uptake in macrophages.** (A) α2MG-MCs were added to RAW 246.7 cells, up to 10 capsules/cell for 24h, along with a positive control (LPS, 2μg/ml) and vehicle (PBS), prior of adding MTT solution. Formazan production was measured with a fluorescence plate reader. Data are mean ± SEM of 3 independent experiments (One way ANOVA, Bonferroni post-test, **p<0.01 *vs.* vehicle). (B) Time-dependent uptake of MCs in macrophages, up to 24h. Cell-associated fluorescence corresponding to the amount of phagocyted capsules has being quantified with a plate reader. Data are mean ± SEM of 3 independent experiments (One way ANOVA, Bonferroni post-test, *p<0.05, **p<0.01, ***p<0.001 *vs.* vehicle, dotted line).


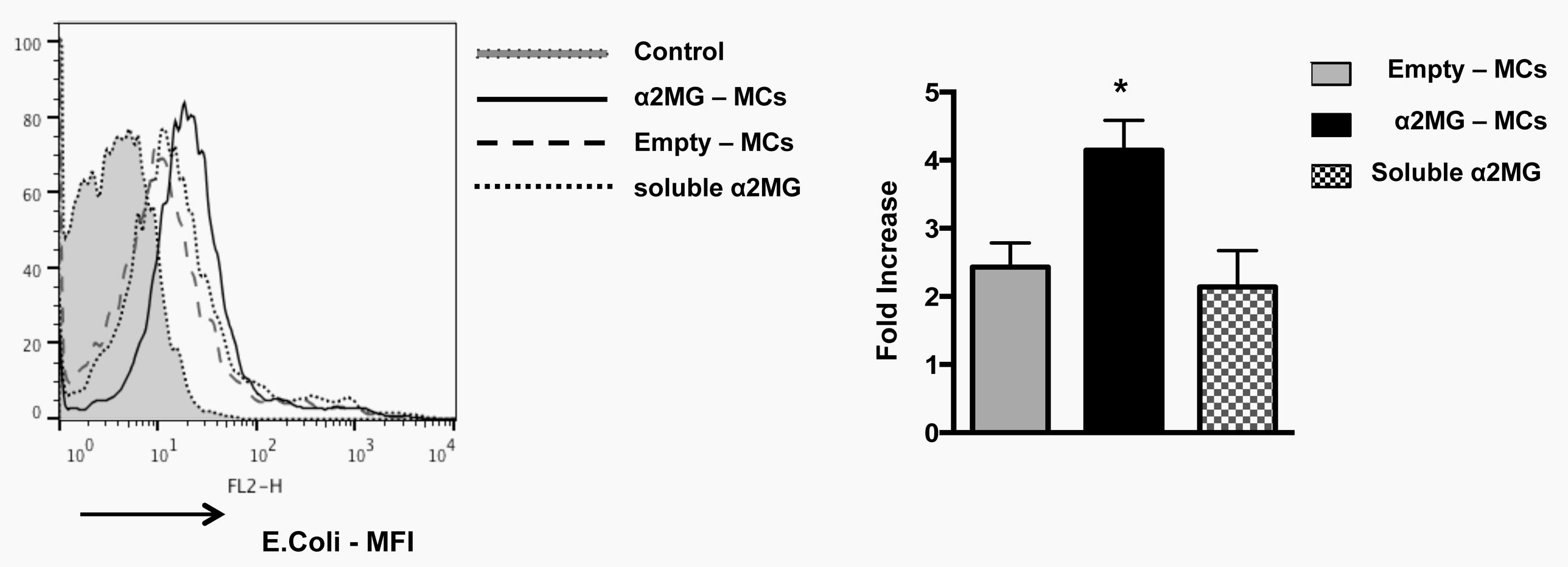


**Supplementary Figure 2. α2MG-microcapsules promote pH-sensitive-*E.Coli* uptake in macrophages.** Human macrophages were incubated with vehicle, α2MG-MCs, empty capsule (1x10^5^) or soluble α2MG (94ng) for 24h prior of application of pH-sensitive *E. Coli* particles (1mg/ml, 30min). Data are mean ± SEM of 5 different human macrophage preparations, and are expressed as fold increase over control. Unpaired t-test, *p<0.05 *vs.* α2MG-MCs).

**
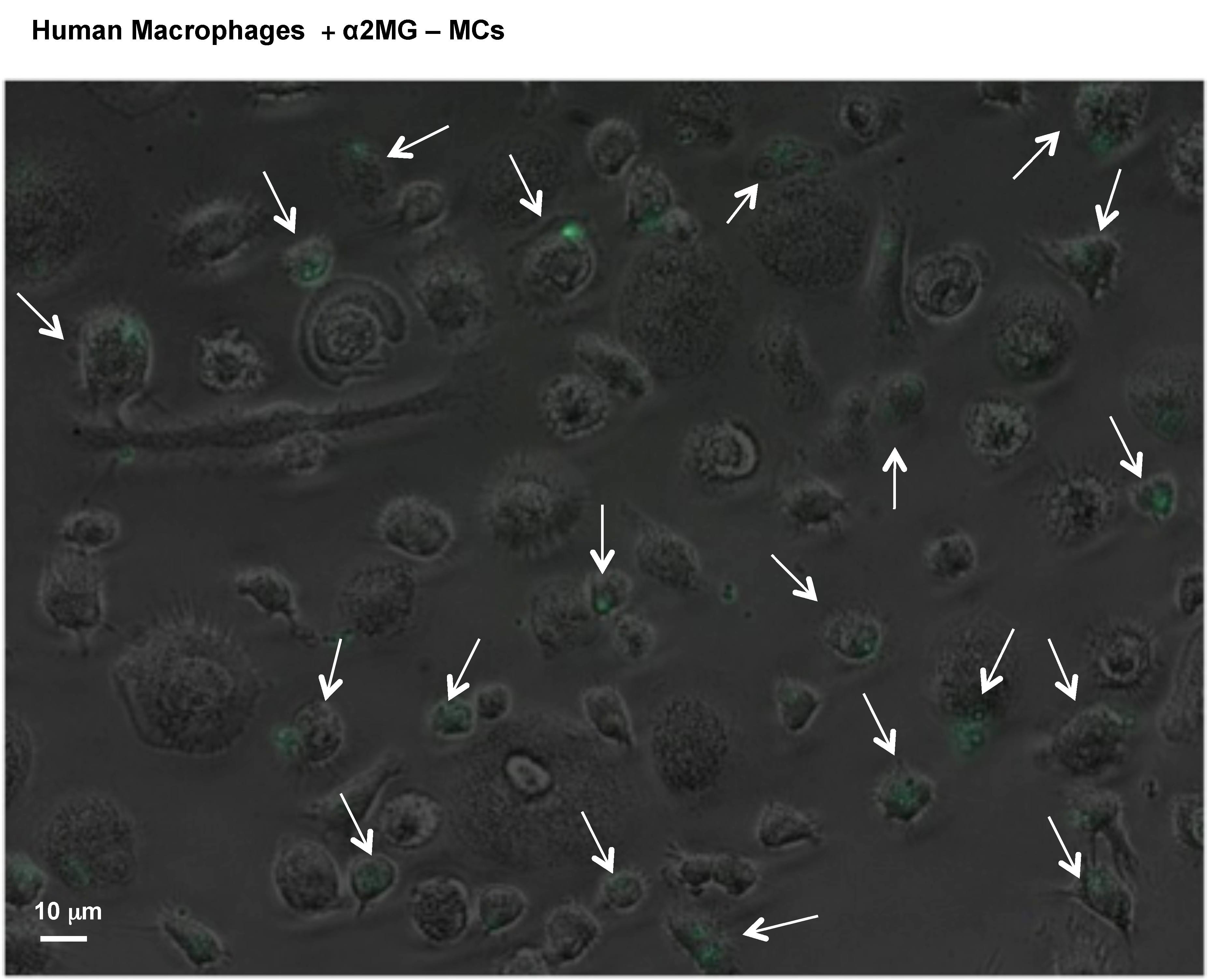
**

**Supplementary Figure 3. α2MG-microcapsules interaction with human monocyte-derived macrophages (MDM).** Human macrophages were incubated with α2MG-MCs for 24h prior to visualizing their update: a great disparity was observed across the cells. White arrows indicate cells that have engulfed a large number of capsules.


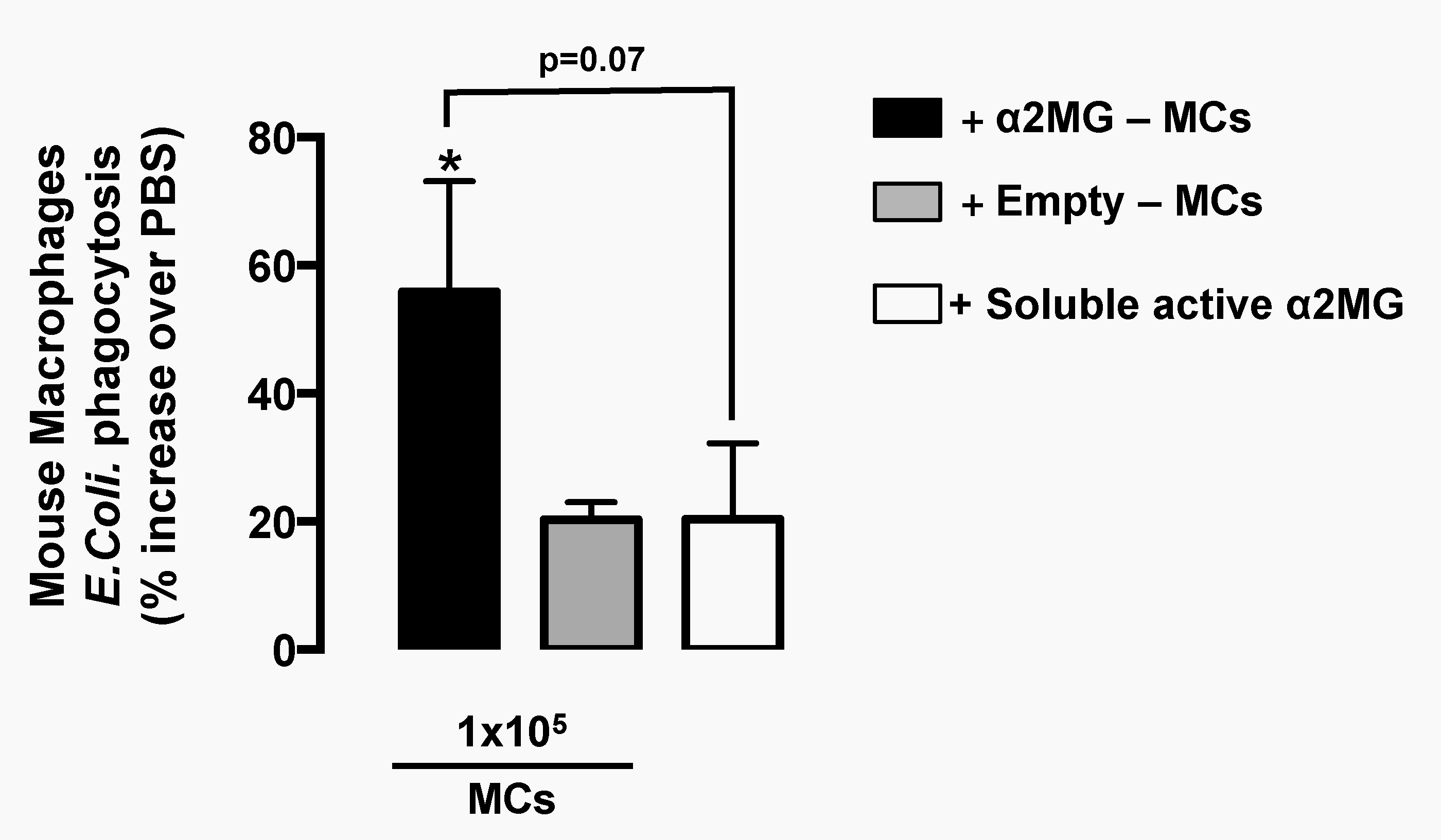


**Supplementary Figure 4. α2MG-microcapsules** **promote phagocytosis in biogel-elicited murine macrophages.** Biogel-elicited mouse macrophages were incubated with vehicle (PBS), α2MG-MCs or empty MCs (1x10^5^/well) for 24h before addition of fluorescent *E. Coli* (1mg/ml, 1h) particles. Soluble α2MG (94ng/well) was used for comparative purposes. The number of phagocytized particles was determined with a fluorescence plate reader. Data are mean ± SEM of 3 independent experiments (Student’s *t* test, *p<0.05 *vs.* vehicle).

**
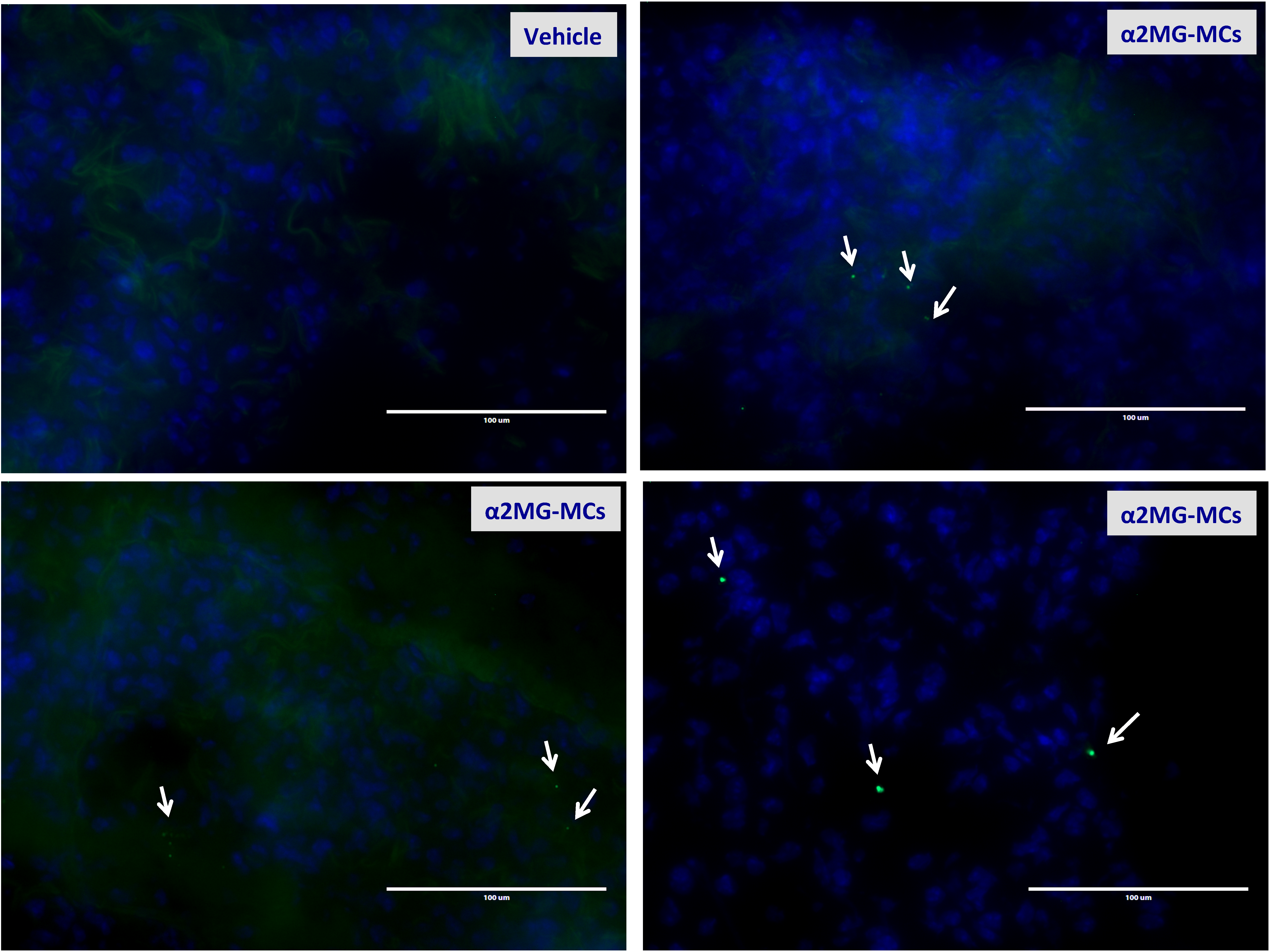
**

**Supplementary Figure 5. α2MG-microcapsules** **localize into the lung tissue once injected intravenously.** Mice were treated i.v. with α2MG-MCs or PBS and organs collected 24 h later. MCs were visualized in the lung tissue sections selectively in mice treated with α2MG-MCs. Images are representative of three distinct analyses.

**References**

[1] Volodkin DV, Petrov AI, Prevot M, Sukhorukov GB. Matrix polyelectrolyte microcapsules: new system for macromolecule encapsulation. Langmuir : the ACS journal of surfaces and colloids. 2004;20:3398-406.

[2] Dalli J, Norling LV, Montero-Melendez T, Federici Canova D, Lashin H, Pavlov AM, et al. Microparticle alpha-2-macroglobulin enhances pro-resolving responses and promotes survival in sepsis. EMBO molecular medicine. 2014;6:27-42.

[3] Amoruso A, Bardelli C, Gunella G, Fresu LG, Ferrero V, Brunelleschi S. Quantification of PPAR-gamma protein in monocyte/macrophages from healthy smokers and non-smokers: a possible direct effect of nicotine. Life sciences. 2007;81:906-15.

[4] Jaffe EA, Nachman RL, Becker CG, Minick CR. Culture of human endothelial cells derived from umbilical veins. Identification by morphologic and immunologic criteria. The Journal of clinical investigation. 1973;52:2745-56.

[5] Norling LV, Dalli J, Flower RJ, Serhan CN, Perretti M. Resolvin D1 limits polymorphonuclear leukocyte recruitment to inflammatory loci: receptor-dependent actions. Arteriosclerosis, thrombosis, and vascular biology. 2012;32:1970-8.

[6] Gobbetti T, Le Faouder P, Bertrand J, Dubourdeau M, Barocelli E, Cenac N, et al. Polyunsaturated fatty acid metabolism signature in ischemia differs from reperfusion in mouse intestine. PloS one. 2013;8:e75581.
